# Dietary fat intake and risk of esophageal carcinoma: a meta-analysis of observational studies

**DOI:** 10.18632/oncotarget.21462

**Published:** 2017-10-03

**Authors:** Du He, Xue Huang, Zai-Ping Wang, Dian Chen, Jun Chen, Chun-Yan Duan

**Affiliations:** ^1^ Department of Oncology, The Central Hospital of Enshi Autonomous Prefecture, Enshi 445000, China; ^2^ Enshi Clinical College of Wuhan University, Enshi 445000, China

**Keywords:** fat intake, esophageal carcinoma, risk, meta-analysis

## Abstract

Dietary fat intake is potentially associated with the onset of esophageal carcinoma (EC), but evidence from observational studies has remained unclear. This study aimed to evaluate the role of fat intake in the development of esophageal adenocarcinoma (EAC) and esophageal squamous cell carcinoma (ESCC). A systematic search was conducted in PubMed and Web of Science to identify all relevant studies. Study-specific relative risks (RR) for the highest versus the lowest intake categories and 95% confidence intervals (CI) were pooled using a random-effects model. Seventeen case-control studies (2058 EAC cases, 1581 ESCC cases and 11696 controls) and two prospective cohort studies (494, 978 participants and 630 EAC cases and 215 ESCC cases) were identified. In EAC, the RRs (95% CI) were 1.69 (1.14–2.50) for total fat intake, 1.88 (1.28–2.77) for saturated fat (SFA) intake, 1.04 (0.86–1.27) for polyunsaturated fat (PUFA) intake and 1.70 (1.01–2.84) for monounsaturated fat (MUFA) intake. In ESCC, the RRs (95% CI) were 1.12 (0.84–1.51) for total fat, 1.38 (0.91–2.08) for SFA, 0.95 (0.55–1.62) for PUFA and 1.04 (0.65–1.66) for MUFA. In conclusion, total fat, SFA and MUFA intake were associated with EAC risk, but fat intake showed no significant association with ESCC risk. Large-scale prospective cohort studies are needed to confirm our findings.

## INTRODUCTION

Esophageal carcinoma (EC) is one of the most common cancers around the world, with two main types of squamous cell carcinoma (ESCC) and adenocarcinoma (EAC) [1]. As about 455,800 EC cases and 400,200 deaths occurred per year, it is necessary to identify the etiology or risk factors, and prevent the disease from the source. Multiple factors were reported in relation with the pathogenesis of EC, including smoking, alcohol, obesity, low consumption of fruits and vegetables, and high-temperature drinking [2, 3]. In recent years, the incidence of ESCC decreased in North America and Europe due to the reduction in alcohol and tobacco use [4–6]. Meanwhile, the incidence of EAC has been increasing in western countries including America, Australia, France and Britain [7]. It might contribute to the increasing obesity which increases the risk for gastroesophageal reflux disease (GERD), Barrett's esophagus (BE) and subsequent EAC development. Thus, EC might be prevented through a healthy lifestyle including a healthy diet [8]. For dietary food, high intakes of vegetables and fruit have been reported in inverse association with both EAC and ESCC risk [9, 10]. Yogurt intake was inversely associated with ESCC risk, while red and processed meat intake were related with increased risk of ESCC [11, 12]. For dietary nutrients, dietary intake of fiber, vitamin C and folate showed an inverse association with both EAC and ESCC [13–15]. However, the role of fat intake is controversial in the pathogenesis of EC, and no meta-analyses have concentrated on this. Therefore, we conducted a meta-analysis of observational studies to evaluate the role of fat intake in the development of EC. Furthermore, as the subtypes of EAC and ESCC were different in the pathogenesis, we analyzed EAC and ESCC respectively.

## MATERIALS AND METHODS

### Search strategy

The databases of PubMed and Web of Science were searched for relevant studies published up to 6th September 2017, using the key words including: (“diet*” OR “fat*” OR “nutrition”) AND (“esophageal” OR “esophagus” OR “upper gastrointestinal tract”) AND (“cancer” OR “carcinoma” OR “tumor” OR “malignancy”). Studies in languages other than English or Chinese were excluded. Moreover, we also reviewed the references of related studies and reviews for undetected studies.

### Study selection and exclusion

Two authors (D.H. and X.H.) reviewed the studies independently. The inclusion criteria were as follows: (i) case-control or cohort-based study design; (ii) evaluated the role of fat intake in the development of EAC and ESCC respectively; (iii) presented relative risk (RR), odds ratio (OR), or hazard ratio (HR) estimates with 95% confidence intervals (CI). The exclusion criteria were as follows: abstracts without full-text, reviews, case reports, pediatric, and animal studies.

### Data extraction and quality assessment

Two authors (D.H. and Z.P.W.) extracted the data by a standardized collection form. All differences were resolved by discussion with a third author (J.C.). In each study, the following information was extracted: first author, publication year, location, study design, EC subtype, numbers of cases and controls (or participants), study period, exposure assessment, adjusted factors. The Newcastle–Ottawa Scale (NOS) was used to assess the methodological quality of included studies [16].

### Statistical analysis

As the incidence of EC was less than 10%, OR and HR could be roughly regarded as the RR in this study [17]. As EAC and ESCC were different in the process of carcinogenesis, they were analyzed respectively in this study. To evaluate the risk of high fat intake, we pooled the risk estimates for the highest versus lowest categories of intake. A random-effects model was used as the pooling method, which considered both within-study and between-study variation. The heterogeneity between studies was estimated by *Q* test and *I*^2^ statistic, and *I*^2^ > 50% represented substantial heterogeneity [18]. Subgroup analyses were conducted on the main confounders to evaluate the stability of main results. For those with more than five included studies, Egger's test was used to detect publication bias. If publication bias was present, the “trim and fill” strategy was used to adjust the funnel plot and re-computed the result [19]. All statistical analyses were performed with STATA version 12.0 software (StataCorp, College Station, TX, USA), and *P* values < 0.05 were considered statistically significant.

## RESULTS

### Study characteristics

**Figure 1:**
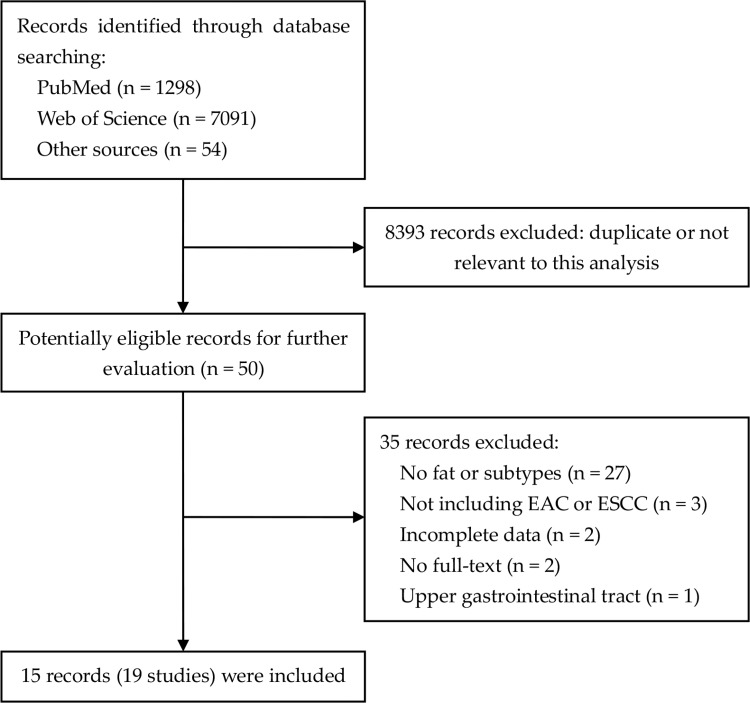
Flowchart of literature search

The search strategy resulted in 8493 records: 1298 from PubMed, 7091 from Web of Science and 54 through other sources (Figure 1; Supplementary Table 1). After excluding duplicated and irrelevant records, 15 records (19 studies) were included in this meta-analysis [20–34]. The characteristics of the included studies were listed in Supplementary Table 4. Tzonou et al, Mayne et al, O’Doherty et al and Lagergren et al classified the results by EC subtypes, namely EAC and ESCC. Thus, these studies were divided into two separate reports. Among these 19 studies, 17 were case-control designed with a total of 2794 EC cases and 11696 controls, while two studies were prospective cohort designed with a total of 494,978 participants and 845 cases. Nine studies focused on EAC with a total of 2058 cases, while ten on ESCC with a total of 1581 cases. Food frequency questionnaires (FFQ) were used to measure fat intake in all studies, which contained various food items and intake frequency. The results in most studies were statistically adjusted for certain factors, like age, gender, drinking, smoking and energy intake. In quality assessment, the included studies had an average score of 6.83 (Supplementary Table 2; Supplementary Table 3).

### Total fat intake and EC risk

**Figure 2:**
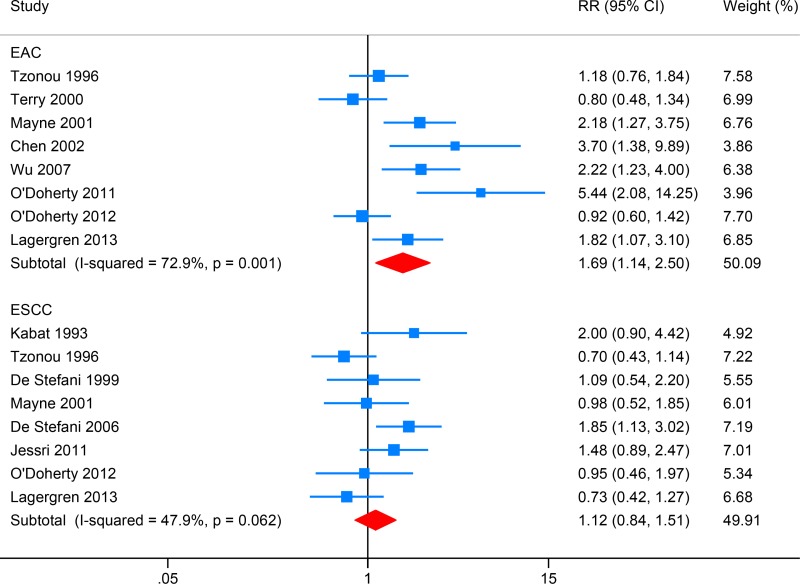
Forest plot (random-effects model) for the meta-analysis of total fat intake and risk of esophageal carcinoma

Eight studies investigated the association between total fat intake and EAC risk, while eight focused on ESCC (Figure 2). High intake of total fat intake was associated with increased EAC risk (RR: 1.69, 95% CI: 1.14–2.50; *I*^2^ = 72.9%), and Egger's test detect obvious publication bias (*P* = 0.014). After introducing the “trim and fill” method to adjust this bias, the overall effect size was still significant (RR: 1.31, 95% CI: 1.13–1.49). Total fat intake showed no significant association with ESCC risk (RR: 1.12, 95% CI: 0.84–1.51; *I*^2^ = 47.9%), and Egger's test detect no obvious publication bias (*P* = 0.817).

### Saturated fat (SFA) intake and EC risk

**Figure 3:**
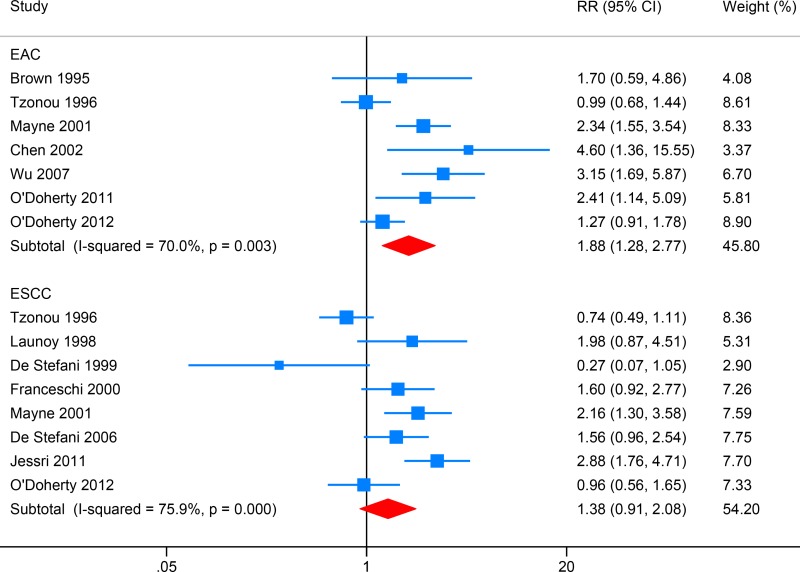
Forest plot (random-effects model) for the meta-analysis of saturated fat (SFA) intake and risk of esophageal carcinoma

Seven studies reported the association between SFA intake and EAC risk, while eight focused on ESCC (Figure 3). High SFA intake was associated with increased EAC risk (RR: 1.88, 95% CI: 1.28–2.77; *I*^2^ = 70.0%), and Egger's test detect no obvious publication bias (*P* = 0.134). SFA intake showed no significant association with ESCC risk (RR: 1.38, 95% CI: 0.91–2.08; *I*^2^ = 75.9%), and Egger's test detect no obvious publication bias (*P* = 0.738).

### Polyunsaturated fat (PUFA) intake and EC risk

**Figure 4:**
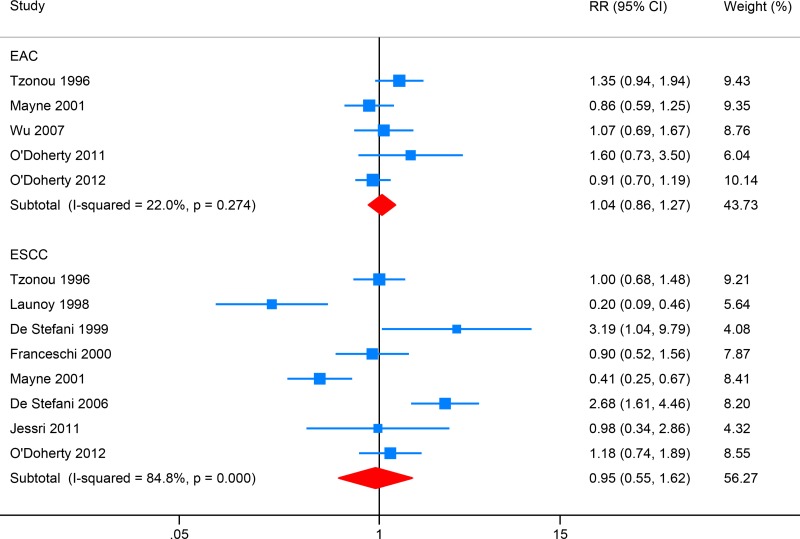
Forest plot (random-effects model) for the meta-analysis of polyunsaturated fat (PUFA) intake and risk of esophageal carcinoma

Five studies investigated the association between total fat intake and EAC risk, while eight focused on ESCC (Figure 4). High PUFA intake showed no significant association with the risk of EAC (RR: 1.04, 95% CI: 0.86–1.27; *I*^2^ = 22.0%) or ESCC (RR: 0.95, 95% CI: 0.55–1.62; *I*^2^ = 84.8%). Egger's test detect no obvious publication bias (*P* = 0.274; *P* = 0.920).

### Monounsaturated fat (MUFA) intake and EC risk

**Figure 5:**
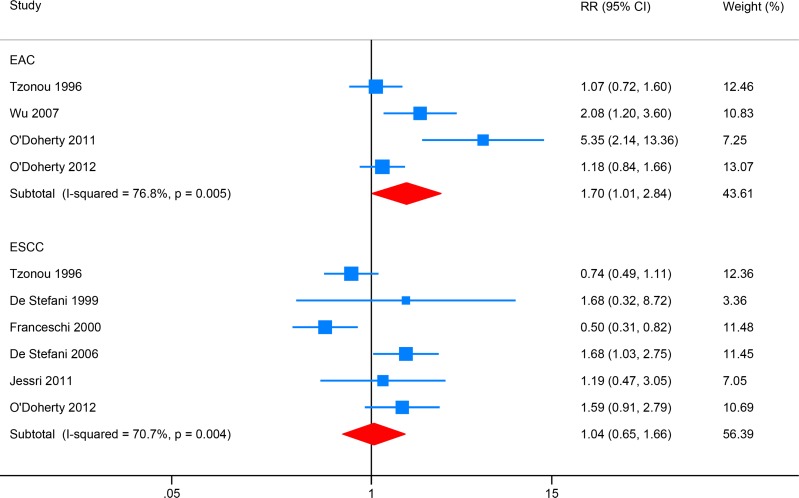
Forest plot (random-effects model) for the meta-analysis of monounsaturated fat (MUFA) intake and risk of esophageal carcinoma

Four studies investigated the association between total fat intake and EAC risk, while six focused on ESCC (Figure 5). High MUFA intake was associated with increased EAC risk (RR: 1.70, 95% CI: 1.01–2.84; *I*^2^ = 76.8%). MUFA intake showed no significant association with ESCC risk (RR: 1.04, 95% CI: 0.65–1.66; *I*^2^ = 70.7%), and Egger's test detect no obvious publication bias (*P* = 0.531).

### Subgroup analysis

Subgroup analyses were conducted on study design, cohort, adjustment of energy intake and smoking for EAC and ESCC respectively, and we found no significant difference between subgroups, except for those subgroups with only one included study (Tables 1 and 2).

**Table 1:** Subgroup analysis of fat intake and risk of esophageal adenocarcinoma

Subgroup	Total fat			SFA			PUFA			MUFA		
	*N*	RR (95% CI)	*I*^2^	*N*	RR (95% CI)	*I*^2^	*N*	RR (95% CI)	*I*^2^	*N*	RR (95% CI)	*I*^2^
Study design												
Population-based	7	1.82 (1.15–2.90)	75.8	6	2.15 (1.47–3.16)	56.5	4	0.95 (0.79–1.15)	0	3	2.13 (1.01–4.51)	81.1
Hospital-based	1	1.18 (0.76–1.84)	-	1	0.99 (0.68–1.44)	-	1	1.35 (0.94–1.94)	-	1	1.07 (0.72–1.60)	-
Cohort												
Caucasian	8	1.69 (1.14–2.50)	72.9	7	1.88 (1.28–2.77)	70.0	5	1.04 (0.86–1.27)	22	4	1.70 (1.01–2.84)	76.8
Asian	-	-	-	-	-	-	-	-	-	-	-	-
Energy intake adjustment												
Yes	7	1.57 (1.06–2.33)	73.0	6	1.75 (1.19–2.58)	70.6	5	1.04 (0.86–1.27)	22	4	1.70 (1.01–2.84)	76.8
No	1	3.70 (1.38–9.98)	-	1	4.60 (1.36–15.55)	-	-	-	-	-	-	-
Smoking adjustment												
Yes	7	1.57 (1.06–2.33)	73.0	6	1.75 (1.19–2.58)	70.6	5	1.04 (0.86–1.27)	22	4	1.70 (1.01–2.84)	76.8
No	1	3.70 (1.38–9.98)	-	1	4.60 (1.36–15.55)	-	-	-	-	-	-	-

SFA, saturated fat; PUFA, polyunsaturated fat; MUFA, monounsaturated fat; N, number of included studies; RR, relative risk; CI, confidence interval.

**Table 2:** Subgroup analysis of fat intake and risk of esophageal squamous cell carcinoma

Subgroup	Total fat			SFA			PUFA			MUFA		
	*N*	RR (95% CI)	*I*^2^	*N*	RR (95% CI)	*I*^2^	*N*	RR (95% CI)	*I*^2^	*N*	RR (95% CI)	*I*^2^
Study design												
Population-based	3	0.86 (0.60–1.23)	0.0	2	1.45 (0.65–3.21)	0.0	2	0.70 (0.25–1.96)	89.4	1	1.59 (0.91–2.79)	-
Hospital-based	5	1.30 (0.87–1.95)	59.1	6	1.34 (0.79–2.28)	79.4	5	1.06 (0.54–2.08)	84.2	5	0.94 (0.56–1.59)	70.1
Cohort												
Caucasian	7	1.07 (0.77–1.49)	50.3	7	1.23 (0.83–1.84)	69.0	7	0.94 (0.53–1.69)	87.0	5	1.03 (0.61–1.74)	76.2
Asian	1	1.48 (0.89–2.47)	-	1	2.88 (1.76–4.71)	-	1	0.98 (0.34–2.86)	-	1	1.19 (0.47–3.05)	-
Energy intake adjustment												
Yes	7	1.06 (0.79–1.43)	46.6	8	1.38 (0.91–2.08)	75.9	8	0.95 (0.55–1.62)	84.8	6	1.04 (0.65–1.66)	70.7
No	1	2.00 (0.90–4.42)	-	-	-	-	-	-	-	-	-	-
Smoking adjustment												
Yes	8	1.12 (0.84–1.51)	47.9	8	1.38 (0.91–2.08)	75.9	8	0.95 (0.55–1.62)	84.8	6	1.04 (0.65–1.66)	70.7
No	-	-	-	-	-	-	-	-	-		-	-

SFA, saturated fat; PUFA, polyunsaturated fat; MUFA, monounsaturated fat; N, number of included studies; RR, relative risk; CI, confidence interval.

## DISCUSSION

Several meta-analyses have reported the association between dietary factors and EC risk. In the meta-analyses of Li et al and Liu et al, vegetables and fruit intake were inversely related with the risk of EAC and ESCC [9, 10]. This might contribute to the rich content in fiber and vitamin C, which were also inversely related with EAC and ESCC risk [14, 15]. However, not dietary factors played the same role in the development EAC and ESCC, as for obvious difference in the pathogenesis. Just like the consumption of hot food and beverages, it was associated with an increased risk of ESCC, but the relationship was not significant for EAC [35]. Thus, it was necessary to evaluate the role of fat intake in EAC and ESCC respectively.

As the main source of fat, the meta-analysis of Salehi et al also evaluated the association between meat intake and EC risk [36]. Total meat, fish and poultry intake were not associated with EAC or ESCC. Red meat intake was associated with an increased risk of ESCC, but not significant for EAC. Processed meat intake was associated with EAC risk, but not significant for ESCC. It's interesting that not all kinds of meat were related with EC risk, although most of them are rich in fat. Moreover, the role of meat intake was different between EAC and ESCC. It was hypothesized that fat intake might also be associated with EC risk, and play different roles between EAC and ESCC. Several meta-analyses also evaluated the association between fat intake and the risk of multiple cancers. Total fat intake was significantly associated with the risk of ovarian cancer and breast cancer [37, 38]. SFA intake was significantly associated with gastric cancer, while PUFA intake was inversely related [39]. No obvious association was found between fat intake and the risk of pancreatic cancer and prostate cancer [40, 41]. These indicated that the risk by fat intake might vary from cancer types and fat types.

In our study, we found a positive association between total fat, SFA and MUFA intake and EAC risk, but no association between fat intake and ESCC risk. It suggested an association between fat intake and EC risk, especially EAC, and the result was consistent with previous experimental studies. In the mice subcutaneously implanted with EAC cells (OE33), the cells displayed increased growth rates, proliferation, and metabolic activity relative to tumors of EAC when the mice were fed high-fat diet [42]. The mechanism remains unclear and complex. Several studies have focused on the ability of adipose tissue to function as an endocrine organ [43]. Excessive adipose tissues caused by high fat intake could release more adipokines, of which some have tumorigenic effects. The well-known adipokine of leptin has been shown to increase proliferation and survival of EAC cell line *in vitro* [44].

This meta-analysis study has several strengths. First, to our knowledge, this is the first meta-analysis of observational studies to identify the association between fat intake and EC risk. Second, EAC and ESCC were analyzed respectively considering obvious difference in the pathogenesis. Third, subgroup analysis was conducted to identify potential confounders, and the results were stable. There were also a few limitations in this study. First, the inclusion of case-control studies might introduce certain bias, such as recall bias, which might potentially lead to differential misclassification of various types of exposure, and exaggerate or weaken the effect estimates. Second, not all potential confounders were adjusted in each study. Third, high heterogeneity existed in the meta-analyses.

In conclusion, total fat, SFA and MUFA intake were associated with EAC risk, but fat intake showed no significant association with ESCC risk. Large-scale prospective cohort studies are needed to confirm our findings.

## SUPPLEMENTARY MATERIALS TABLES






