# Supplementary material for: Dietary fat intake and risk of esophageal carcinoma: a meta-analysis of observational studies
Source: Oncotarget. 2017 Oct 3;8(58):99049–56. doi: 10.18632/oncotarget.21462 (PMC5716790; doi:10.18632/oncotarget.21462)
Supplement: Supplementary file 1 [file oncotarget-08-99049-s001.pdf]

Dietary fat intake and risk of esophageal carcinoma: a meta-analysis of observational studies

SUPPLEMENTARY MATERIALS

Supplementary Table 1: Lists of 35 excluded studies. See Supplementary_Table_1

Supplementary Table 2: Methodological qualtiy of prospective cohort studies inclued in the meta-analysis

First author, year	Representative of exposed cohort	Selection of the unexposed cohort	Ascertainment of exposure	Outcome of interest not present at start of study	Control for important factor or additional factor	Assessment of outcome	Follow-up long enough for outcomes to occur	Adequacy of follow-up of cohorts	Using an energy-adjusted model	Score
O'Doherty 2012	☆	☆	–	☆	☆	☆	☆	☆	☆	8

Supplementary Table 3: Methodological qualtiy of case-control studies inclued in the meta-analysis

First author, year	Adequate definition of cases	Representativeness of cases	Selection of control subjects	Definition of control subjects	Control for important factor or additional factor	Exposure assessment	Same method of ascertainment for all subjects	Nonresponse rate	Using an energy-adjusted model	Score
Graham 1990	☆	☆	☆	☆	☆	–	☆	–	–	6
Kabat 1993	☆	☆	☆	☆	☆	–	☆	–	–	6
Hu 1994	☆	☆	☆	☆	☆	–	☆	–	–	6
Brown 1995	☆	☆	☆	☆	☆	–	☆	–	☆	7
Tzonou 1996	☆	☆	☆	☆	☆	–	☆	–	☆	7
Launoy 1998	☆	☆	☆	☆	☆	–	☆	–	☆	7
De Stefani 1999	☆	☆	☆	☆	☆	–	☆	–	☆	7
Franceschi 2000	☆	☆	☆	☆	☆	–	☆	–	☆	7
Terry 2000	☆	☆	☆	☆	☆	–	☆	–	☆	7
Mayne 2001	☆	☆	☆	☆	☆	–	☆	–	☆	7
Chen 2002	☆	☆	☆	☆	☆	–	☆	–	–	6
Tavani 2003	☆	☆	☆	☆	☆	–	☆	–	☆	7
De Stefani 2006	☆	☆	☆	☆	☆	–	☆	–	☆	7
Wu 2007	☆	☆	☆	☆	☆	–	☆	–	☆	7
Jessri 2011	☆	☆	☆	☆	☆	–	☆	–	☆	7
O'Doherty 2011	☆	☆	☆	☆	☆	–	☆	–	☆	7
Lagergren 2013	☆	☆	☆	☆	☆	–	☆	–	☆	7

Supplementary Table 4: Characteristics of included studies. See Supplementary_Table_4
