# Supplementary material for: Dietary fat intake and risk of esophageal carcinoma: a meta-analysis of observational studies
Source: Oncotarget. 2017 Oct 3;8(58):99049–56. doi: 10.18632/oncotarget.21462 (PMC5716790; doi:10.18632/oncotarget.21462)
Supplement: Supplementary file 2 [file oncotarget-08-99049-s002.docx]

**Supplementary Table 1: Lists of 35 excluded studies.**

| **Study** | **Title** | **Excluding reason** |
| --- | --- | --- |
| Hormozdiari 1975 | Dietary factors and esophageal cancer in the Caspian Littoral of Iran | No fat or subtypes |
| Graham 1990 | Nutritional epidemiology of cancer of the esophagus | Not including EAC or ESCC |
| Chen 1992 | Dietary trace elements and esophageal cancer mortality in Shanxi, China. | No fat or subtypes |
| Rogers 1993 | A case-control study of element levels and cancer of the upper aerodigestive tract | No fat or subtypes |
| Tavani 1993 | Risk factors for esophageal cancer in women in northern Italy | No fat or subtypes |
| Gao 1994 | Risk factors for esophageal cancer in Shanghai, China. II. role of diet and nutrients | Incomplete data |
| Hu 1994 | Risk factors for oesophageal cancer in northeast China | Not including EAC or ESCC |
| Brown 1995 | Adenocarcinoma of the esophagus: role of obesity and diet | No full-text |
| Sichieri 1996 | Diet and mortality from common cancers in Brazil an ecological study | No fat or subtypes |
| Zhang 1997 | Adenocarcinomas of the esophagus and gastric cardia: the role of diet | Upper gastrointestinal tract |
| Brown 1998 | Dietary factors and the risk of squamous cell esophageal cancer among black and white men in the United States | Incomplete data |
| Bosetti 2000 | Food groups and risk of squamous cell esophageal cancer in northern Italy | No fat or subtypes |
| Cheng 2000 | A case-control study of oesophageal adenocarcinoma in women: a preventable disease | No fat or subtypes |
| Terry 2000 | Antioxidants and cancers of the esophagus and gastric cardia | No fat or subtypes |
| Wolfgarten 2001 | Coincidence of nutritional habits and esophageal cancer in Germany | No full-text |
| Bollschweiler 2002 | Vitamin intake and risk of subtypes of esophageal cancer in Germany | No fat or subtypes |
| Tavani 2003 | n-3 polyunsaturated fatty acid intake and cancer risk in Italy and Switzerland | Not including EAC or ESCC |
| Lee 2005 | Adenocarcinomas of the esophagus and gastric cardia: the role of diet | No fat or subtypes |
| Cai 2006 | Dietary selenium intake, aldehyde dehydrogenase-2 and X-ray repair cross-complementing 1 genetic polymorphisms, and the risk of esophageal squamous cell carcinoma | No fat or subtypes |
| Rossi 2007 | Flavonoids and risk of squamous cell esophageal cancer | No fat or subtypes |
| Dong 2008 | Dietary supplement use and risk of neoplastic progression in esophageal adenocarcinoma a prospective study | No fat or subtypes |
| Navarro 2008 | Food group intake and risk of subtypes of esophageal and gastric cancer | No fat or subtypes |
| Carman 2009 | Vitamin E intake and risk of esophageal and gastric cancers in the NIH-AARP Diet and Health Study | No fat or subtypes |
| Islami 2009 | Patterns of food and nutrient consumption in northern Iran, a high-risk area for esophageal cancer | No fat or subtypes |
| Lipworth 2009 | Dietary vitamin D and cancers of the oral cavity and esophagus | No fat or subtypes |
| Malekshah 2010 | Vitamin deficiency in Golestan Province, northern Iran a high-risk area for esophageal cancer | No fat or subtypes |
| Murphy 2010 | Dietary antioxidant and mineral intake in humans is associated with reduced risk of esophageal adenocarcinoma but not reflux esophagitis or Barrett's esophagus | No fat or subtypes |
| Navarro 2011 | Principal component analysis of dietary and lifestyle patterns in relation to risk of subtypes of esophageal and gastric cancer | No fat or subtypes |
| Bravi 2012 | Dietary patterns and the risk of esophageal cancer | No fat or subtypes |
| Ibiebele 2013 | Dietary antioxidants and risk of Barrett's esophagus and adenocarcinoma of the esophagus in an Australian population | No fat or subtypes |
| Sharp 2013 | Intakes of dietary folate and other B vitamins are associated with risks of esophageal adenocarcinoma, Barrett's esophagus, and reflux esophagitis | No fat or subtypes |
| Tang 2014 | Fruit and vegetable consumption and risk of esophageal cancer: a case-control study in north-west China | No fat or subtypes |
| Xiao 2014 | Intakes of folate, methionine, vitamin B6, and vitamin B12 with risk of esophageal and gastric cancer in a large cohort study | No fat or subtypes |
| Hashemian 2015 | Dietary intake of minerals and risk of esophageal squamous cell carcinoma results from the Golestan Cohort Study | No fat or subtypes |
| Dai 2016 | Dietary magnesium, calcium, magnesium ratio and risk of reflux oesophagitis, Barrett's oesophagus and oesophageal adenocarcinoma: a population-based case-control study | No fat or subtypes |

EC: esophageal carcinoma. ESCC: esophageal squamous cell carcinoma.
