# Supplementary material for: Dietary fat intake and risk of esophageal carcinoma: a meta-analysis of observational studies
Source: Oncotarget. 2017 Oct 3;8(58):99049–56. doi: 10.18632/oncotarget.21462 (PMC5716790; doi:10.18632/oncotarget.21462)
Supplement: Supplementary file 3 [file oncotarget-08-99049-s003.doc]

**Supplementary Table 4: Characteristics of included studies.**

| **Study** | **Location** | **Design** | **Cancer Subtype** | **Participants (Cases/Total)** | **Study Period** | **Assessment** | **Adjusted Factors** |
| --- | --- | --- | --- | --- | --- | --- | --- |
| Kabat 1993 | America | Hospital-based case-control | ESCC | 75/4129 | 5 years before diagnosis | FFQ | Smoking, alcohol, age, sex, race, education, hospital, remaining dietary factors |
| Brown 1995 | America | Population-based case-control | EAC | 162/847 | 5 years before diagnosis | FFQ | Age, area, smoking, liquor, income, calories from food, BMI |
| Tzonou 1996 (1) | Greece | Hospital-based case-control | EAC | 56/168 | 1 year before diagnosis | FFQ | Gender, age, birthplace, schooling, height, analgesics, coffee, alcohol, smoking, energy intake |
| Tzonou 1996 (2) | Greece | Hospital-based case-control | ESCC | 43/129 | 1 year before diagnosis | FFQ | Gender, age, birthplace, schooling, height, analgesics, coffee, alcohol, smoking, energy intake |
| Launoy 1998 | France | Hospital-based case-control | ESCC | 208/607 | The previous year before interview | FFQ | Age, interviewer, smoking, alcohol, total energy intake |
| De Stefani 1999 | Uruguay | Hospital-based case-control | ESCC | 82/330 | 1 year before symptom onset | FFQ | Age, sex, residence, urban/rural status, education, hospital, family history, BMI, smoking, alcohol, mate ingestion, total energy intake, vegetables and fruits |
| Franceschi 2000 | Italy | Hospital-based case-control | ESCC | 304/1047 | 2 years before diagnosis | FFQ | Age, sex, residence, education, physical activity, BMI, smoking, alcohol, non-alcohol energy |
| Terry 2000 | Sweden | Population-based case-control | EAC | 185/1000 | 20 years before interview | FFQ | Age, gender, BMI, total energy, alcohol, fruit and vegetable, smoking, antacids |
| Mayne 2001 (1) | America | Population-based case-control | EAC | 282/969 | 3-5 years before diagnosis | FFQ | Sex, site, age, race, proxy status, income, education, BMI, smoking, alcohol, energy intake |
| Mayne 2001 (2) | America | Population-based case-control | ESCC | 206/893 | 3-5 years before diagnosis | FFQ | Sex, site, age, race, proxy status, income, education, BMI, smoking, alcohol, energy intake |
| Chen 2002 | America | Population-based case-control | EAC | 124/573 | At least 3 years before diagnosis | FFQ | Age, sex, BMI |
| De Stefani 2006 | Uruguay | Hospital-based case-control | ESCC | 234/1170 | 5 years before interview | FFQ | Age, sex, residence, urban/rural status, birthplace, education, BMI, smoking, alcohol, mate consumption, total energy intake |
| Wu 2007 | America | Population-based case-control | EAC | 206/1514 | 1 year before diagnosis | FFQ | Age, sex, race, birthplace, education, smoking, BMI, reflux, vitamin use, total calories |
| Jessri 2011 | Iran | Hospital-based case-control | ESCC | 47/143 | 1 year before diagnosis | FFQ | Age, sex, reflux, BMI, smoking, physical activity, education, energy intake |
| O'Doherty 2011 | Ireland | Population-based case-control | EAC | 224/480 | 5 years before interview | FFQ | Age, sex, smoking, BMI, job, education, energy intake, fruit and vegetable, alcohol, Hp infection, nonsteroidal anti-inflammatory drug, gastroesophageal reflux symptoms, location |
| O'Doherty 2012 (1) | America | Prospective cohort | EAC | 630/494978 | An average follow-up of 9.7 years | FFQ | Age, sex, non-alcohol energy intake, BMI, education, ethnicity, alcohol, smoking, diabetes, physical activity, fruit and vegetable intake, red meat intake |
| O'Doherty 2012 (2) | America | Prospective cohort | ESCC | 215/494978 | An average follow-up of 9.7 years | FFQ | Age, sex, non-alcohol energy intake, BMI, education, ethnicity, alcohol, smoking, diabetes, physical activity, fruit and vegetable intake, red meat intake |
| Lagergren 2013 (1) | Sweden | Population-based case-control | EAC | 189/1009 | 2-5 years before interview | FFQ | Sex, age, reflux, BMI, smoking, alcohol, education, total energy intake |
| Lagergren 2013 (2) | Sweden | Population-based case-control | ESCC | 167/987 | 2-5 years before interview | FFQ | Sex, age, reflux, BMI, smoking, alcohol, education, total energy intake |

EAC, esophageal adenocarcinoma; ESCC, esophageal squamous cell carcinoma; FFQ, food frequency questionnaire; BMI, body mass index.
